# Economic Burden of Heart Failure: Investigating Outpatient and Inpatient Costs in Abeokuta, Southwest Nigeria

**DOI:** 10.1371/journal.pone.0113032

**Published:** 2014-11-21

**Authors:** Okechukwu S. Ogah, Simon Stewart, Obinna E. Onwujekwe, Ayodele O. Falase, Saheed O. Adebayo, Taiwo Olunuga, Karen Sliwa

**Affiliations:** 1 Division of cardiology, Department of Medicine, University College Hospital, Ibadan, Nigeria; 2 Soweto Cardiovascular Research Unit, Faculty of Health Sciences, University of the Witwatersrand, Johannesburg, South Africa; 3 Hatter Institute for Cardiovascular Research in Africa and Institute of Infectious Disease and Molecular Medicine, Faculty of Health Sciences, University of Cape Town, Cape Town, South Africa; 4 Department of Health Administration and Management, Faculty of Health Sciences and Technology, University of Nigeria, Enugu Campus, Enugu State, Nigeria; 5 Department of Medicine, Federal Medical Centre, Abeokuta, Nigeria; 6 National Health and Medical research Council Centre of Research Excellence to Reduce, Inequality in Heart Disease, Melbourne, Australia; University of Milan, Italy

## Abstract

***Background:*** Heart failure (HF) is a deadly, disabling and often costly syndrome world-wide. Unfortunately, there is a paucity of data describing its economic impact in sub Saharan Africa; a region in which the number of relatively younger cases will inevitably rise.

***Methods:*** Heath economic data were extracted from a prospective HF registry in a tertiary hospital situated in Abeokuta, southwest Nigeria. Outpatient and inpatient costs were computed from a representative cohort of 239 HF cases including personnel, diagnostic and treatment resources used for their management over a 12-month period. Indirect costs were also calculated. The annual cost per person was then calculated.

***Results:*** Mean age of the cohort was 58.0±15.1 years and 53.1% were men. The total computed cost of care of HF in Abeokuta was 76, 288,845 Nigerian Naira (US$508, 595) translating to 319,200 Naira (US$2,128 US Dollars) per patient per year. The total cost of in-patient care (46% of total health care expenditure) was estimated as 34,996,477 Naira (about 301,230 US dollars). This comprised of 17,899,977 Naira- 50.9% ($US114,600) and 17,806,500 naira −49.1%($US118,710) for direct and in-direct costs respectively. Out-patient cost was estimated as 41,292,368 Naira ($US 275,282). The relatively high cost of outpatient care was largely due to cost of transportation for monthly follow up visits. Payments were mostly made through out-of-pocket spending.

***Conclusion:*** The economic burden of HF in Nigeria is particularly high considering, the relatively young age of affected cases, a minimum wage of 18,000 Naira ($US120) per month and considerable component of out-of-pocket spending for those affected. Health reforms designed to mitigate the individual to societal burden imposed by the syndrome are required.

## Introduction

Heart failure (HF) has recently emerged as a global health problem [1]. It is a highly symptomatic syndrome that affects 2–3% of the population in high income countries especially in people above the age of 65 years [2], [3]. Approximately 15 million (out of 900 million) Europeans and 5.8 million (out of 300 million) Americans are affected by HF [4], [5]. The burden of HF has increased due to increasing elderly population as well as improved survival of patients with risks of HF such as acute myocardial infarction, hypertension and diabetes mellitus [6], [7]. In response, strategies for the effective management of the disease in the outpatient setting have been developed and applied [5]. This has led to improved survival in some countries [4], [8], [9]. In high-income countries, the economic burden of HF is high because it is associated with frequent hospital admissions [10], [11]. Moreover, management of HF places significant financial burden on patients, their families/care-givers and, as indicated, society as a whole [10], [12], [13].

Until recently, little was known about the emerging problem of non-communicable forms of HF supplementing traditional pathways to the syndrome in sub-Saharan Africa. Contemporary studies from South Africa[14], [15] and Nigeria[16]–[19] have built on historical reports to demonstrate that the aetiology, natural history and indeed case profile of HF (i.e. more women and younger individuals affected in the prime of their life) is different from high-income countries. As such, HF is now responsible for 7–10% of medical admissions in the region [16], [19], [20]. Significantly, given its potential enormous cost implications (both related to direct health care costs and economic burden on affected individuals and their families) there is virtually no data on the economic burden of HF in sub-Saharan African countries and major populaces such as Nigeria.

### Study Aims & Objectives

The aim of this study, therefore, was to determine the consumption of health care resources for the treatment of HF in Abeokuta, Nigeria and to estimate overall healthcare costs associated with its management in a large and representative cohort of affected cases. Data were used to estimate the annual cost of HF in the region to inform health care policy towards efficient use of resources to mitigate the individual to societal impact of the syndrome.

## Material and Methods

### Ethics Statement

This study was approved by the Federal Medical Center Abeokuta and University of Witwatersrand Health Research Ethics Review Committees and all the participants provided informed consent in writing, in accordance with the Declaration of Helsinki [21].

### Study site

The study was undertaken at the Federal Medical Centre, Abeokuta, Ogun state, southwest Nigeria. The state has a population of about 3,751,140 inhabitants [22]. Abeokuta is the state capital and has a population of about a million people [23]. As described previously [19], Federal medical center Abeokuta is a tertiary institution that receives referrals from primary and secondary health facilities within and outside the state. All the patients that were discharged alive were given monthly appointments for clinical review as well as for refill of their medications. Patients pay out of pocket and many may not be able to buy medication that will last for longer period. Home based nursing care is not in existence in the city and in many parts of the country. Patients are often taken care of by their relations. Healthcare cost in the city of Abeokuta and in most parts of Nigeria is generally borne by the patient through out of pocket payment payments.

Only a small proportion of the Nigerian population has access to social health insurance [24]–[26]. However, there exist very strong family bonding and extended family systems where poor patients are assisted by their wealthy or well-to-do family members. The limited coverage of social health insurance in Nigeria is a very big challenge to the achievement of universal health coverage and health care delivery in the country.

### Study design and sample

As described previously [19], the Abeokuta HF registry was a hospital based, prospective, observational registry that ran from January 2009 to December 2010 which was established to determine the current clinical profile of HF in the city as well as to assess typical clinical outcomes and healthcare costs (the focus of this report) associated with HF. A more detailed description of the registry has been documented [19]. Briefly, all cases of HF presenting to the hospital were captured into the database. Private health facilities, primary health centres as well as health workers were sensitized on the existence of the registry with good response. To a large extent, the sample was fully representative of the population. During the period, HF was responsible for 9% of total medical admission [19] a figure that is consistent with equivalent reports from other parts of Africa [14], [18], [20].

### Data collection

A specific data extraction proforma was developed and used to obtain data on resource consumption in those cases enrolled in the Abeokuta HF registry. The clinical diagnostic criteria employed in the diagnosis of HF have been described elsewhere [19]. Clinical diagnoses were coded using the International Classification of Diseases (ICD-10) coding system [27]. The proforma was used to extract the following direct outpatient and inpatient cost categories as shown in the textbox below.

### Health Care Expenditure

#### Direct Health Care Costs

The inpatient costs include the cost of medical and nursing care, cost of investigations and drugs **(** **Table 1** **)**. It also includes the cost of surgery and procedures as well transportation to and from the hospital. Personnel costs include the opportunity cost of medical care, nursing as well as ancillary support by other health workers. The wages of these health workers were derived from the Federal Government of Nigeria salary scales. This included their basic salary; call duty allowance, hazard allowance and housing allowance. We did not capture cost associated with purchase of medications over the counter, cost of alternative medical care which is common in Africa, aids, home modifications etc. We assume that these categories are likely to represent a very small percentage of the total costs identified from this study. We also did not capture capital costs.

**Table 1:** Shows summary of the cost components.

Cost component
A.
B. In-patient care
**i.** Direct cost
Hospital care (Medical consultation)
Nursing care, utility fees, accommodation and feeding and ward consumables including oxygen)
Medications
Medications
Surgery and procedures
Transportation
Surgery and procedures
ii. Indirect cost- the days of lost work (productivity loss)
B. Out-patient care
**i.** Direct cost
Clinic visits
Laboratory investigations
Medications
Transportation
ii. Indirect cost-the days of lost work (productivity loss)

#### Indirect Health Care Costs

For indirect costs, the days of lost work (productivity loss) were calculated and the minimum wage used to monetize them.

#### In-patient cost

The standard costing table (unit costs) of the Federal Medical Center, Abeokuta for the year 2010 was used for computing the cost of consultations, hospital admissions, medical consumables, medical investigations, and drug therapy **(** **Table 1** **)**. It was assumed that the cost of single hospital admission covers the cost of treatment in the emergency room as well as in the medical ward. The cost of medications was taken as a whole. We assumed that the cost of medications had remained unchanged throughout the year.

#### Out-patient costs

This was also grouped into direct and indirect costs. All the patients that were discharged alive were given monthly appointments for clinical review as well as for refill of their medications **(** **Table 1** **)**.

### Specific costs

#### Cost of drugs

The cost of drugs was based on the hospital's price list (which includes the tender prices plus a 10% mark up or dispensing fee). The frequency of use of the various categories of drugs was based on the findings from the HF registry. The most frequent dosage was used for cost calculation. We did not account for increase or reduction in dose of the drugs during the course of treatment. We assumed that substitution of one class or brand of medication occurred without additional costs.

#### Cost of Laboratory and diagnostic tests

The cost of all laboratory and diagnostic tests (haematological, biochemical, microbiological, chest radiography, electrocardiography and echocardiography) were based on the price list of the hospital in 2010. The rate of consumption of these items was also garnered from the HF registry.

#### Cost of Surgery and Procedures

The cost of surgeries and procedures were obtained from the Lagos State University Teaching Hospital (LASUTH), as well the Reddington multi-specialist hospital, Lagos where majority of the surgeries and procedures were performed [28], [29]. The surgeries and procedures include valve surgeries, coronary angioplasty/stenting, pericardiectomies etc. Total cost was multiplied by the frequency of the surgeries and procedures.

#### Estimation of indirect costs

The human capital approach was used to estimate the indirect cost. The average annual earning of the patients was estimated based on their occupational group. These were used to calculate their average daily earnings. The product of the working days lost and average daily earnings provided the productivity losses associated with HF in the study and these were assigned monetary values.

### Cost analyses

All available costs (as detailed above) associated with in-patient care, out-patient care as well as the opportunity costs associated with HF management in the city were computed for the year 2010. An annual, prevalence based approach was employed in estimating the cost of the resources used for the management HF [10]–[12], [30]. Healthcare costs were then expressed in the local currency-Naira, (and converted to US Dollar [$US] at a rate of 150 Nigerian Naira to $US1 in 2010).

## Results

Outpatient and inpatient costs were computed from a representative cohort of 239 HF cases over a 12-month period for the year 2010. **Table 2** provides a summary of the study cohort used to derive all HF-related health care activities. Mean age was 58.0±15.1 years, 46.9% were female and around one third were aged ≤55 years and in the prime of their potential working life.

**Table 2:** Sociodemographic profile of the HF subjects seen in 2010.

Variable	Value
Gender	
Male (n/%)	127(53.1)
Female (n/%)	112(46.9)
Mean age (years)	58.0(15.1)
Median age(years)	60
Age range (years)	
<24	8(3.3)
25–34	15(6.3)
35–44	21(8.8)
45–54	40(16.7)
55–64	59(24.7)
65–74	63(26.4)
> = 75	33(13.8)
Marital status	
Single	32(13.4)
Married	168(70.3)
Widow/widower	5(2.1)
Separated/Divorced	34(14.2)
Educational level	
No formal education	88(36.8)
Primary (< = 6 years)	60(25.1)
Secondary (6–12 years)	44(18.4)
Post-Secondary/University/Postgraduate (>12years)	47(19.7)
Occupation	
Unemployed	20(8.4)
Unskilled labour	146(61.1)
Skilled labour	18(7.5)
Professional	24(10.0)
Pensioner/Retiree	31(13.0)
Place of residence	
Within Abeokuta city	138(57.7)
Outside Abeokuta but within Ogun State	53(22.2)
Outside Abeokuta and outside Ogun State	48(20.1)
Monthly Income of subjects in Naira(Median value)	
Unemployed*	18000(120USD)
Unskilled labour	90,000(600USD)
Skilled labour	240,000(1600USD)
Professional	480,000(3200USD)
Pensioner/Retiree	330,000(2200USD)
Aetiology of HF	
Hypertensive HF	195(81.6)
Dilated Cardiomyopathy	14(5.9)
Pericardial Diseases	8(3.3)
Rheumatic Heart Disease	4(1.7)
Others	4(1.7)
Co-morbidities	
Hypertension	185(77.4)
Osteoarthritis	49(20.9)
Atrial fibrillation	37(15.5)
Diabetes Mellitus	24(10.0)
Chronic Obstructive pulmonary disease	7(2.9)
Others	6(2.4)

*Allocated minimum wage in the country.

### Cost of components of in-patient care

#### Cost of hospital care

The following were computed for the subjects: medical consultation (N300/day), nursing care (N200/day), utility fees(N400/day), accommodation and feeding (N900/day) and average cost of ward consumables/patient/admission (N3500/patient)The estimated cost of hospital care for the year was about 5.5 million naira (about $U36,500).

#### Cost of laboratory investigations

The laboratory investigations considered in the costing is as shown in **Table 3(A)**.The estimated cost was approximately 3.8 million naira (about $US28, 000).

**Table 3:** Cost of investigations (In-patient).

**A. In-patient Investigations**					
**Investigation**	**Cost per test**	**Percent**	**Number**	**Cost (Naira)**	**Cost (US Dollars)**
Urinalysis	200	88	225	45000	300.0
Full blood count	1350	96	229	309150	2061.0
Blood sugar	400	96	229	91600	610.7
Electrolyte and Urea	2500	96	229	572500	3816.7
Lipid profile	3500	65	155	542500	3616.7
Electrocardiography	2500	96	229	572500	3816.7
Echocardiography	5000	92	220	1100000	7333.3
Chest X-Ray	2000	96	229	458000	3053.3
Cardiac enzyme	5000	0.8	2	10000	66.7
INR	3500	10	24	84000	560.0
HIV screening	500	46	111	55500	370.0
Liver function tests	3000	50	120	360000	2400.0
**Total Cost**				**3840750**	**28005.0**
**B. Out-patient investigations**					
**Investigation**	**Cost per test**	Percent*	**Number**	**Cost (Naira)**	**Cost (US Dollars)**
Electrocardiography	2500	96	197	492500	3283.3
Electrolyte and Urea	2500	96	197	492500	3283.3
Total	5000	96	394	985000	6566.7

Note: Cost based on the hospital costing list for 2009.

Note: Calculation based on those that survived, it is assumed that same proportion on admission.

performed these investigations during follow up.

#### Cost of medications

This includes cost of standard drugs for the management of HF, as well as other drugs such as antituberculous drugs used for the management of T.B pericarditis. The estimated cost of drugs was N6, 565,527 (about $US43770) **(see** **Table 4** **).**

**Table 4:** In-patient cost of medications.

Drug	Route	Dose	Dosing	Unit cost (Naira)	Cost per daily dose(Naira)	Total Cost(Naira)	Number of subjects	Total cost in US Dollars
Frusemide	IV	40 mg	3	20	60	13620	227	90.8
Frusemide	Oral	40 mg	3	5	15	3405	227	22.7
Amlodipine	Oral	5 mg	1	15	15	615	41	4.1
Lisinopril	Oral	10 mg	1	15	15	3330	222	22.2
Spironolactone	Oral	25 mg	1	10	10	2030	203	13.5
Digoxin	Oral	0.125 mg	1	5	5	1015	203	6.8
Carvedilol	Oral	6.25 mg	1	25	25	550	22	3.7
Hydrallazine	Oral	25 mg	1	5	5	90	18	0.6
Isosorbide dinitrate	Oral	10 mg	1	220	220	3960	18	26.4
Heparin (LMW)	SC	40 mg	1	1350	1350	209250	155	1395.0
Warfarin	Oral	2.5 mg	1	40	40	1120	28	7.5
Atorvastatin	Oral	10 mg	1	180	180	7380	41	49.2
Amiodarone	Oral	200 mg	2	20	40	200	5	1.3
Antibiotics (Augmentin)	IV	600 mg	3	600	1800	399600	222	2664.0
Antibiotics (Augmentin)	Oral	625 mg	2	170	340	75480	222	503.2
Prednisolone	Oral	30 mg	1	30	30	7170	239	47.8
Anti-tuberculous therapy	Oral	Multiple drugs	1	60	60	480	8	3.2
Aspirin	Oral	75 mg	1	2	2	208	104	1.4
Total						729503		4863.4
Grand Total						6565527		43770.2

Source: Federal Medical Centre, Abeokuta costing list, Median length of hospital stay = 9 days, 1 US Dollar  = 150 naira (2010).

#### Cost of Surgery/Procedures

As stated earlier, we estimated the cost of surgery/procedures for the few patients who were able to afford them. This was estimated to cost N1, 092, 000 (about $US72800) – see **Table S1.**

#### Cost of transportation

This was calculated to cost N216, 600 (about $US1, 444) see **Table S2.**

#### Indirect in-patient care cost

This was estimated to cost N17, 806,500 Naira ($US118, 710) – see **Table 5A** **.**

**Table 5:** Indirect cost (In-patient/outpatient).

**A. Indirect in-patient care cost**							
Occupation	Monthly Income	Daily income	Daily income(Dollars)	No of Subjects	Total Income lost/day(Naira)	Income lost (Naira)**	Income lost (Dollar)**
Unemployed*	18000	900	6	20	18000	162000	1080
Unskilled	90000	4500	30	146	657000	5913000	39420
Skilled	240000	12000	80	18	216000	1944000	12960
Professional	480000	24000	160	24	576000	5184000	34560
Pensioners	330000	16500	110	31	511500	4603500	30690
Total	1158000	57900	386	239	1978500	17806500	118710
**B. Indirect out-patient care cost**							
Occupation	Monthly Income	Daily income	Daily income(Dollars)	No of Subjects	Total Income lost/day(Naira)	Income lost (Naira)**	Income lost (Dollar)**
Unemployed*	18000	900	6	14	12600	151200	1008
Unskilled	90000	4500	30	130	585000	7020000	46800
Skilled	240000	12000	80	15	180000	2160000	14400
Professional	480000	24000	160	21	504000	6048000	40320
Pensioners	330000	16500	110	25	412500	4950000	33000
Total	1158000	57900	386	205	1694100	20329200	135528

Note: Income loss was based on 5 working days in a week, **Income loss was based on median Los of 9 days.

Note: *Assigned the minimum wage in Nigeria, Income loss was based on 5 working days in a week,

Because most mortality occurred early, it was assumed that 205 subjects completed follow up, 1dollar  = 150Nigerian Naira.

### Cost of components of out-patient care

#### Out-patient clinic visit

This was based on the cost of medical consultations (N250/visit) and nursing/ancillary services (N200/visit). The calculated total cost was N29, 700 ($US198 US dollar) – see **Table S3.**

#### Cost of out-patient investigations

This was calculated for subjects who survived initial hospital admission. It was assumed that the proportion of subjects who had these tests during admission were also able to have them done during the follow up period. It was estimated to cost N985, 000 ($US 6567 – see **Table 3B** **.**

#### Cost of outpatient medications

This is shown in **Table S4.** It was estimated to cost N9, 154,168 ($US61, 028).

#### Cost of outpatient transportation

This constituted a greater proportion of the out-patient care cost. It was calculated as N9, 717, 000 ($US 64,780) – see **Table S5.**

#### Indirect out-patient care cost

This was estimated to cost about 20,329,200 Naira ($US135, 528) – see -**Table 5B** **.**

### Summary of in-patent and out-patient care cost

**Figure 1:**
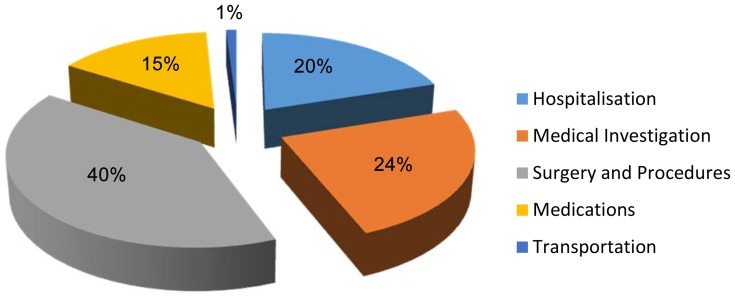
Components of direct cost (In-patient).

The total cost of in-patient care was estimated as N34, 996, 477 ($US301, 230). This comprised of N17, 899, 977 (50.9%, $US114, 600) and N17, 806,500 (49.1%, $US118, 710) for direct and in-direct costs respectively. Direct costs were responsible for 61% of in-patient care costs. About 40% of the direct cost was due to surgery/procedures. Hospitalization, medical investigations, drug therapy, and transportation accounted for 20%, 24%, 15%, and 1% respectively, of costs –see **Table 6** **(A) and** **Figure 1** **.**

**Table 6:** Summary of various aspects of costs of in-patient/out-patient care.

**A. Summary of various aspects of costs of in-patient care**		
Item	Total cost (in Naira)	Total Cost in US Dollars
Hospitalization	5475100	36500.7
Investigations	3840750	28005
Medications	6565527	43770.2
Surgery and procedures	10920000	72800
Transportation	216600	1444
Total Direct Cost	17189977	114600
Total Indirect Cost	17806500	118710
**Grand total**	**34996477**	**233310**
**B. Summary of various aspects of costs of out-patient care**		
Item	Total cost (in Naira)	Total Cost in US Dollars
Clinic visit	1107000	7380
Investigations	985000	6567
Medications	9154168	61028
Transportation	9717000	64780
Total Direct cost	20963168	139754
Indirect cost	20329200	135528
**Grand total**	**41292368**	**275282**

**Figure 2:**
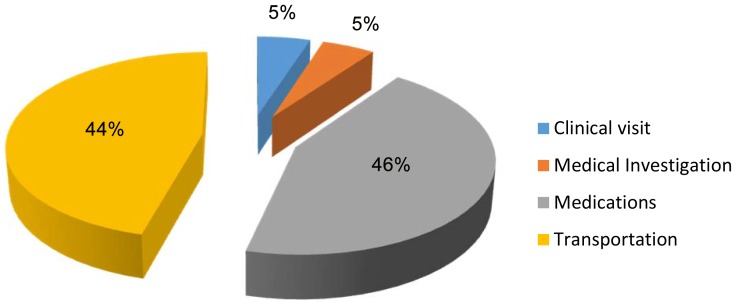
Components of direct cost (out-patient).

**Table 6(B)** summarizes the components and total estimated cost of out-patient care. It was estimated as N41, 292, 368 ($US 275,282 UD dollars). Direct and in-direct costs were N20, 963,168 ($US139, 754) and N20, 329,200 ($US135, 528) respectively, constituting 51% and 49% of total out-patient care costs. Transportation, medications, clinic visits and medical investigations contributed 46%, 44%, 5% and 5% respectively to these costs - see **(** **Figure 2** **)**.

### Total cost

**Figure 3:**
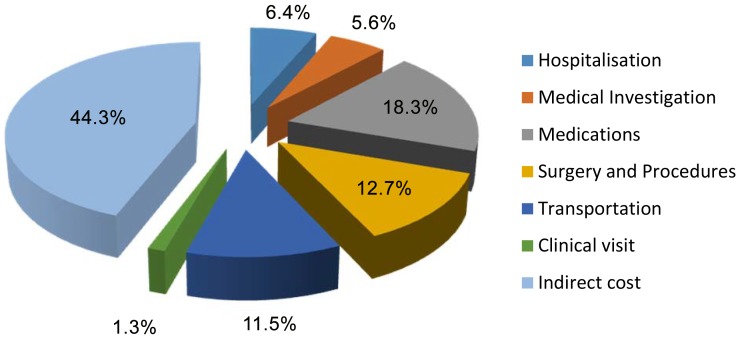
Percentage contribution of different components to total cost.

The total estimated cost of care of HF in Abeokuta for the year 2010 was N76, 288,845 ($US508, 595) translating to N319, 200 ($US2, 128) per patient per year. The proportional contribution of in-patient and out-patient cost were 46% and 54% respectively. The contribution of various components to the total cost is shown in **Figure 3**.

## Discussion

To our knowledge this is the first systematic attempt at estimating the cost of HF in Nigeria; a major populace of sub-Saharan Africa. We have calculated the cost from an individual perspective whilst calculating the total cost per annum overall from a societal perspective. Effort was made to capture all the HF patients in the city of Abeokuta during the study period. All cases of HF presenting to the two major hospitals in the city were captured into the database. Private health facilities, primary health centres as well as health workers were sensitized on the existence of the registry with good response. To a large extent, the sample was representative of the population. During the period, HF was responsible for 9% of total medical admission similar to earlier report from other parts of Africa [14], [18], [20]. The main findings of the study are - 1. The total cost of HF or cost per patient per year is enormous considering the context of a developing economy where out-of-pocket expenses is the main means of health care financing;2. Furthermore the estimated cost of HF was fairly distributed between in-patient and out-patient care; 3. A large proportion of direct cost for in-patient care was due to surgery and medical procedures and; 4. Out-patient medications and transportation was responsible for 90% of direct cost of out-patient care. The huge cost of HF has been well documented by several reports both at the individual and population levels. Data from the National Heart and Lung Institute of the United Kingdom shows that the cost of HF per person is approximately 8500 Pounds. At the national level HF costs about 39.2 billion US dollars in America (about 2% of US healthcare budget) [31]. Similar data have been reported from other countries [10], [11] [12], [32].

The distribution of cost of HF in our setting is different from that observed in high income countries [10], [11], [32] but similar to one report from Brazil [12]. There has not been any report from Africa to compare our findings with. The contribution of hospital care cost in these countries ranged from 53–75%. Cost of outpatient care was in the range of 4–31% while the cost of drug therapy was between 6–8% of the total cost of care. The pattern of heart diseases as well as level of technological development influences the mode of care as well as utilization of sophisticated and expensive medical equipment, procedures and consumables which are obviously needed for the care of HF patients. The consumption of these is higher in high income countries than in our setting. This is clearly shown by the impact of surgery/procedures on the cost of hospital care. The few cases (out of the many that needed this) that had surgery for valvular diseases or coronary interventions escalated the hospital care cost in our study.

Other determinants of hospital cost will also include length of hospital stay [10], [13]. (in which our report is similar to European data but longer than the mean length of hospital stay in the USA) Cost of management of co-morbidities has also been shown to account for higher cost of hospitalization in high income countries. In one report on Medicare claims, HF accounted for only15% of the total in-patient cost while 57% were associated with other co-morbid conditions [33]. This may probably be responsible for the huge cost of hospitalization in high income countries compared to our environment.

Due to lack of community based or nurse-led or home care of HF patients in our setting, our patients are given shorter out-patient appointments in order to refill their medications. This is responsible for the high cost attributable to transportation in our report. Because of the younger age of our subjects, HF in our setting is, therefore, associated with longer disability adjusted life years and by extension a huge cost to the society at large.

With the changing demographic and epidemiological landscape in Nigeria coupled with the rising burden of cardiovascular risk factors and non-communicable diseases (especially hypertension) in the country, the rate of HF is predicted to rise if preventive measures are not put in place at all levels. This will put a lot of strain in an already weak health system. The high cost of surgical interventions and procedures is out of the reach of the average Nigerian. Prevention of conditions requiring this mode of care such as rheumatic heart disease, tuberculosis (because of pericarditis) and coronary artery disease should be a priority for the country at large. Furthermore the need for a functional, effective and efficient social health insurance system in the country cannot be overemphasized considering the fact that majority of those afflicted by HF are poor and are not likely to sustain the treatment of their illness for a long time. There is also need to develop community based HF care in the country as this will reduce the cost of outpatient care which is largely contributed by the cost of frequent transport to-and-from the health facility.

### Limitations

This is a hospital based study. Although we tried as much as possible to capture all the HF cases in the city during the study period, there is still the possibility that mild cases may have been missed out especially those that were managed in the out-patient clinic.

We also did not assess cost based on the severity of HF (NYHA class). Studies have shown that those in NYHA class IV were 8–30 times more expensive to manage compared to those in NYHA class II [30], [34], [35]. Age and sex-specific cost analysis was also not carried out. We also did not include the cost of co-morbidities neither did we capture cost due to alternative medicines (which is common in Africa), over the counter purchases, capital cost and indirect cost by care givers as well as the general cost to the society. These are potential areas of research in our community in the future.

### Conclusion

Our data shows the profound impact and importance of HF as a major public health problem in a developing economy like Nigeria that is still battling with communicable diseases. The annual individual cost of HF is high coupled with the fact that of pocket expenses in the country is over 90%. There is therefore need to reduce this expenditure through control of risk factors for HF in the society especially high blood pressure, reduction in hospital as well as out-patient care cost through the development of community HF care programmes in the country.

## Supporting Information

Table S1 **Cost of procedures (In-patient).**
(DOCX)

Table S2 **Cost of transport (In-patients).**
(DOCX)

Table S3 **Cost of clinic visits (Out-patient).**
(DOCX)

Table S4 **Cost of medications (Out-patient).**
(DOCX)

Table S5 **Cost of transport (Out-patient).**
(DOCX)
