# Supplementary material for: Economic Burden of Heart Failure: Investigating Outpatient and Inpatient Costs in Abeokuta, Southwest Nigeria
Source: PLoS One. 2014 Nov 21;9(11):e113032. doi: 10.1371/journal.pone.0113032 (PMC4240551; doi:10.1371/journal.pone.0113032)
Supplement: Table S1 — Cost of procedures (In-patient). (DOCX) [file pone.0113032.s001.docx]

| **Table S1: Cost of procedures (In-patient)** | | | | |
| --- | --- | --- | --- | --- |
|  | | | | |
| **Procedure** | **Number** | **Cost per procedure** | **Total cost(Naira)** | **Cost (US Dollar)** |
| Pericardiocentesis | 11 | 20,000 | 220000 | 1466.7 |
| Pericardiectomy* | 2 | 150,000 | 300,000 | 2000.0 |
| Coronary angioplasty/Stenting* | 2 | 700,000 | 1,400,000 | 9333.3 |
| Valve surgery* | 5 | 1,800,000 | 9000000 | 60000.0 |
| Total |  |  | 10,920,000 | 72800.0 |
| ***Source= Lagos State University Teaching Hospital and Reddington Hospital Lagos (Ref. 28),**  **1 US Dollar = 150 Nigerian Naira,** | | | | |
