# Supplementary material for: Economic Burden of Heart Failure: Investigating Outpatient and Inpatient Costs in Abeokuta, Southwest Nigeria
Source: PLoS One. 2014 Nov 21;9(11):e113032. doi: 10.1371/journal.pone.0113032 (PMC4240551; doi:10.1371/journal.pone.0113032)
Supplement: Table S2 — Cost of transport (In-patients). (DOCX) [file pone.0113032.s002.docx]

| **Table S2: Cost of transport (In-patients)** | | | | |
| --- | --- | --- | --- | --- |
|  |  |  |  |  |
| **Residence** | **Number** | **Mean Cost*** | **Total(Naira)** | **Total Cost (Dollars)** |
| Within Abeokuta | 138 | 250 | 34500 | 230 |
| Outside Abeokuta but within Ogun State* | 53 | 900 | 47700 | 318 |
| Outside Ogun State* | 48 | 2800 | 134400 | 896 |
| Total |  |  | 216600 | 1444 |
| ***Mean Cost= Cost per visit = (to and fro), Source= Transport fair at the city motor park,**  **1 US Dollar= 150 Nigerian Naira** | | | | |
