# Supplementary material for: Economic Burden of Heart Failure: Investigating Outpatient and Inpatient Costs in Abeokuta, Southwest Nigeria
Source: PLoS One. 2014 Nov 21;9(11):e113032. doi: 10.1371/journal.pone.0113032 (PMC4240551; doi:10.1371/journal.pone.0113032)
Supplement: Table S3 — Cost of clinic visits (Out-patient). (DOCX) [file pone.0113032.s003.docx]

| **Table S3: Cost of clinic visits (Out-patient)** | | | | | | |
| --- | --- | --- | --- | --- | --- | --- |
|  | | | | | | |
| **Month (Year_2010)** | **No of admissions** | **Mortality** | **No followed up** | **No of months followed up** | **Cost (Naira)** | **Cost (Dollars)** |
| January | 34 | 0 | 34 | 11 | 4950 | 33 |
| February | 20 | 0 | 20 | 10 | 4500 | 30 |
| March | 16 | 1 | 15 | 9 | 4050 | 27 |
| April | 25 | 5 | 20 | 8 | 3600 | 24 |
| May | 29 | 1 | 28 | 7 | 3150 | 21 |
| June | 20 | 2 | 18 | 6 | 2700 | 18 |
| July | 21 | 1 | 20 | 5 | 2250 | 15 |
| August | 18 | 0 | 18 | 4 | 1800 | 12 |
| September | 12 | 1 | 11 | 3 | 1350 | 9 |
| October | 21 | 2 | 19 | 2 | 900 | 6 |
| November | 9 | 1 | 8 | 1 | 450 | 3 |
| December | 14 | 0 | 14 | 0 | 0 | 0 |
| Total | 239 | 14 | 225 | 66 | 29700 | 198 |
| Assumption= All the mortalities occurred early in the process of follow up,  Cost of medical consultation= N250. Cost of nursing and ancillary services =N200 | | | | | | |
