# Supplementary material for: Economic Burden of Heart Failure: Investigating Outpatient and Inpatient Costs in Abeokuta, Southwest Nigeria
Source: PLoS One. 2014 Nov 21;9(11):e113032. doi: 10.1371/journal.pone.0113032 (PMC4240551; doi:10.1371/journal.pone.0113032)
Supplement: Table S4 — Cost of medications (Out-patient). (DOCX) [file pone.0113032.s004.docx]

| **Table S4: Cost of medications (Out-patient)** | | | | | | | | |
| --- | --- | --- | --- | --- | --- | --- | --- | --- |
|  | | | | | | | | |
| **Drug** | **Route** | **Most frequent dose** | **Most frequent dosing** | **Unit cost (Naira)** | **Cost per daily dose(Naira)** | **Number of subjects** | **Total Cost(Naira)** | **Total cost in US Dollars** |
| Frusemide | Oral | 40mg | 3 | 5 | 15 | 148 | 2220 | 14.8 |
| Amlodipine | Oral | 5mg | 1 | 15 | 15 | 30 | 450 | 3.0 |
| Lisinopril | Oral | 10mg | 1 | 15 | 15 | 203 | 3045 | 20.3 |
| Spironolactone | Oral | 25mg | 1 | 10 | 10 | 203 | 2030 | 13.5 |
| Digoxin | Oral | 0.125mg | 1 | 5 | 5 | 55 | 275 | 1.8 |
| Carvedilol | Oral | 6.25mg | 1 | 25 | 25 | 30 | 750 | 5.0 |
| Hydrallazine | Oral | 25mg | 1 | 5 | 5 | 30 | 150 | 1.0 |
| Isosorbide dinitrate | Oral | 10mg | 1 | 220 | 220 | 30 | 6600 | 44.0 |
| Warfarin | Oral | 2.5mg | 1 | 40 | 40 | 28 | 1120 | 7.5 |
| Atorvastatin | Oral | 10mg | 1 | 180 | 180 | 41 | 7380 | 49.2 |
| Amiodarone | Oral | 200mg | 2 | 20 | 40 | 5 | 200 | 1.3 |
| Centrally acting drugs (Alpha methyldopa) | Oral | 500mg | 2 | 10 | 20 | 36 | 720 | 4.8 |
| Aspirin | Oral | 75mg | 1 | 2 | 2 | 89 | 178 | 1.2 |
| Antidiabetic agent (Metformin) | Oral | 500mg | 2 | 10 | 20 | 22 | 440 | 2.9 |
| Antituberculous therapy | Oral | Multiple drugs | 1 | 40 | 40 | 8 | 320 | 2.1 |
| **Total** |  |  |  |  |  |  | **25878** | **172.5** |
| Grand Total (for 365-9 days) OR 356 days except for antiTB which were for 6 months | | | | |  |  | 9154168 | 61417.1 |
|  | | | | |  |  |  |  |
