# Supplementary material for: Economic Burden of Heart Failure: Investigating Outpatient and Inpatient Costs in Abeokuta, Southwest Nigeria
Source: PLoS One. 2014 Nov 21;9(11):e113032. doi: 10.1371/journal.pone.0113032 (PMC4240551; doi:10.1371/journal.pone.0113032)
Supplement: Table S5 — Cost of transport (Out-patient). (DOCX) [file pone.0113032.s005.docx]

| **Table S5: Cost of transport (Out-patient)** | | | | | |
| --- | --- | --- | --- | --- | --- |
|  |  |  |  |  |  |
| **Residence** | **Number of subjects** | **Mean Cost per visit(to and fro)** | **Mean cost for 12 visits in a year** | **Total Cost in Naira** | **Total Cost in Dollars** |
| Within Abeokuta | 138 | 250 | 3000 | 615000 | 4100 |
| Outside Abeokuta but within Ogun State* | 53 | 900 | 10800 | 2214000 | 14760 |
| Outside Ogun State* | 48 | 2800 | 33600 | 6888000 | 45920 |
| Total | 239 | 3950 | 47400 | 9717000 | 64780 |
| ***Source: Transport fare at the main motor park in the city, 1 US Dollar = 150 Naira,**  **Assumptions: Almost all the patients came for follow up** | | | | | |
